# Childbearing Histories and Midlife Cognition: Accounting for Early Life Factors

**DOI:** 10.1093/geroni/igaf122.425

**Published:** 2025-12-31

**Authors:** Mieke Thomeer, Rin Reczek, Joseph Wolfe, Dee Ferguson, Rui Cao

**Affiliations:** The University of Alabama at Birmingham, Birmingham, Alabama, United States; The Ohio State University, Columbus, Ohio, United States; The University of Alabama at Birmingham, Birmingham, Alabama, United States; The University of Alabama at Birmingham, Birmingham, Alabama, United States; The Ohio State University, Columbus, Ohio, United States

## Abstract

Childbearing histories—for example, parity and age at first birth—matter for the health outcomes of mid- and later-life women. There is growing evidence that childbearing may shape cognitive outcomes. However, previous research is not definitive due to its inability to account for selection. Selection is critical to address given that multiple factors, including socioeconomic background and cognition in adolescence, place women at risk for both specific childbearing experiences and poor cognitive functioning. We analyze the 1979 National Longitudinal Survey of Youth (NLSY79; N = 3,668), a longitudinal nationally representative data set from the United States that began collecting data during respondents’ late teens and early 20s. We estimate the average treatment effects of childbearing histories on midlife memory and cognition using propensity-score matching techniques that incorporate an expansive set of early life factors. In models adjusting for early life covariates, we find that any births, high parity, and early first birth are associated with worse self-reported memory, but not cognitive functioning scores. After addressing the influence of early life factors through matching techniques, the only robust differences relate to any births and early first births with self-reported memory. Selection into specific childbearing experiences partially drives the link between childbearing and cognition at midlife, with cascading implications into later life. We suggest incorporating childbearing and early life factors into models to identify social determinants of memory and cognitive functioning.

